# Pathophysiology of reflux oesophagitis: role of Toll-like receptors 2 and 4 and Farnesoid X receptor

**DOI:** 10.1007/s00428-021-03066-w

**Published:** 2021-03-08

**Authors:** Minna Nortunen, Nina Väkiparta, Katja Porvari, Juha Saarnio, Tuomo J Karttunen, Heikki Huhta

**Affiliations:** 1 Cancer and Translational Medicine Research Unit, Medical Research Center Oulu, University of Oulu and Oulu University Hospital, 90014 Oulu, Finland; 2 Research Unit of Surgery, Anesthesia and Intensive Care, University of Oulu, Oulu, Finland; 3 Department of Surgery, Oulu University Hospital and Medical Research Center Oulu, Oulu, Finland

**Keywords:** Reflux oesophagitis, TLR2, TLR4, FXR

## Abstract

The pathogenesis of gastroesophageal reflux disease (GERD) is not fully understood. It involves the activation of mucosal immune-mediated and inflammatory responses. Toll-like receptors (TLR) 2 and TLR4 are pattern-recognition receptors of the innate immune system; they recognize microbial and endogenous ligands. Farnesoid X receptor (FXR) is a bile acid receptor that regulates the inflammatory response. We aimed to evaluate TLR2, TLR4 and FXR expression patterns in GERD. We re-evaluated 84 oesophageal biopsy samples according to the global severity (GS) score, including 26 cases with histologically normal oesophagus, 28 with histologically mild oesophagitis and 30 with severe oesophagitis. We used immunohistochemistry and in situ hybridization to assess the expression patterns of TLR2, TLR4 and FXR in oesophageal squamous cells. Immunohistochemistry showed that nuclear and cytoplasmic TLR2 was expressed predominantly in the basal layer of normal oesophageal epithelium. In oesophagitis, TLR2 expression increased throughout the epithelium, and the superficial expression was significantly more intensive compared to normal epithelium, *p* <0.01. Nuclear and cytoplasmic TLR4 was expressed throughout the thickness of squamous epithelium, with no change in oesophagitis. FXR was expressed in the nuclei of squamous cells, and the intensity of the expression increased significantly in oesophagitis (*p* <0.05). FXR expression correlated with basal TLR2. In situ hybridization confirmed the immunohistochemical expression patterns of TLR2 and TLR4. In GERD, TLR2, but not TLR4, expression was upregulated which indicates that innate immunity is activated according to a specific pattern in GERD. FXR expression was increased in GERD and might have a regulatory connection to TLR2.

**Supplementary Information:**

The online version contains supplementary material available at 10.1007/s00428-021-03066-w.

## Introduction

In 1935, Winkelstein described a series of patients with heartburn symptoms, ulcerations and inflammation in the distal oesophagus. The diagnosis was oesophagitis, which resulted from tissue damage due to free hydrochloric acid burns and pepsin effects [1]. This conclusion was widely accepted as the pathogenic origin of gastroesophageal reflux disease (GERD). Direct, acid-induced damage to superficial cells was assumed to cause the histological changes characteristic of GERD, including basal cell hyperplasia, intercellular space dilatation, erosion and neutrophil infiltration [2, 3].

In 2009, Souza and co-workers [4] established an experimental rat model of reflux oesophagitis by creating an oesophagoduodenostomy. They showed that inflammatory changes in the oesophagus started from the basal layers of the squamous epithelium. Changes were observed in the submucosa and in the lamina propria within 1 week, but changes in the superficial cells were not detected until 3 weeks post-operatively. Papillary and basal hyperplasia developed before superficial erosion, and lymphocyte infiltration was dominant. The oesophageal epithelium was also exposed to acidified bile salts, which increased the levels of interleukin-8 (IL-8) and IL-1β. Investigators concluded that the damage was induced by a cytokine-mediated inflammatory response that originated in the basal epithelial layers [4].

Later studies have investigated the roles of lymphocytes and cytokines in oesophagitis onset. Dunbar et al. studied a group of patients taking proton-pump inhibitors for treating GERD [5]. When the medication was stopped, the patients experienced a re-induction of reflux oesophagitis. Histologic samples showed that papillary and basal hyperplasia appeared before superficial erosions. Additionally, they detected a significant T lymphocyte infiltration [5]. Various studies in patients with GERD have shown increases in the proinflammatory Th1 cytokines, IL-6, IL-8, IL-10 and IL-1β, in the epithelium, and an inflammatory response mediated by NF-κB [6, 7].

Toll-like receptors (TLR) are pattern-recognition receptors vital to the innate immune system [8–10]. They recognize bacterial, viral and plant motives and can lead to pro- or anti-inflammatory responses. Both TLR2 and TLR4 can activate the intracellular MyD88/NF-κB pathway, which leads to the transcription of proinflammatory cytokines, such as IL-6, IL-8, tumour necrosis factor alpha (TNF-α), IL-10 and type I interferon [10]. We have previously shown that both TLR2 and TLR4 were expressed in normal squamocellular oesophageal epithelium, and expression increased in Barrett’s metaplasia-dysplasia-adenocarcinoma sequence [11], but their participation in oesophageal inflammation is unconfirmed.

The Farnesoid X receptor (FXR) is a nuclear bile acid receptor and regulator. FXR is highly expressed in enterohepatic tissues, but it is also found in vascular walls, adipose tissue and kidneys [12]. FXR controls the homeostasis of intestinal bile acids, lipids and glucose, the extent of inflammation in the intestinal tract and the integrity of the intestinal barrier. In studies with knockout mice [13, 14], FXR was suggested to play a protective role in inflammatory bowel diseases by inhibiting proinflammatory cytokine production. Previous studies on Barrett’s oesophagus found that FXR expression levels rose in oesophagitis [15], but its role might have been to ameliorate inflammation, as proposed by Lian et al., in the context of gastric ulcers [16].

The roles of TLR2 and TLR4 in GERD pathogenesis and their relationship with FXR remain unclear. The present study aimed to evaluate TLR2, TLR4 and FXR expression by immunohistochemistry and in situ hybridization in a representative series of patients with GERD and controls without GERD. We hypothesized that TLR2, TLR4 and FXR activate during gastroesophageal reflux and modulate the mucosal injury.

## Materials and methods

Oesophageal biopsy samples from 84 patients with GERD were obtained from the archives of the Department of Pathology, Oulu University Hospital. The endoscopic samples were collected in 2011–2015 and had been biopsied according to our local protocol with oesophageal biopsies aimed at the Z-line and +2cm. Biopsy samples were divided into three groups, according to the original histological diagnosis: normal oesophagus (*n*=21), mild oesophagitis (*n*=43) and severe oesophagitis (*n*=20). For the assessment of endoscopic degree of oesophagitis, we aimed to retrieve the Los Angeles Classification [17] for each patient from Oulu University Hospital medical records, including the original endoscopy reports and endoscopic footage. However, due to the retrospective setting and insufficient endoscopy reports and footages, LA class was only available in 60% of the final GERD cohort. Therefore, the endoscopic degree of oesophagitis was classified as severe in the presence of ulcerations and mild when superficial erosions, erythema or oedema were seen.

The haematoxylin and eosin stained sections were re-evaluated according to the histological criteria for reflux oesophagitis [18, 19] by an experienced gastrointestinal pathologist (TJK). The elements required for the assessment of reflux oesophagitis, including basal cell layer hyperplasia, papillary elongation, dilatation of intercellular spaces, inflammatory cell infiltration and the presence of healed/active erosions, were evaluated and recorded. The global severity (GS) score, formulated by Mastracci et al. [18] to identify histological reflux oesophagitis, was applied. The GS score cutoff of 0.35 has been reported to have an agreeable correlation with pH monitoring–based diagnosis of GERD. The patients with histological oesophagitis (GS score < 0.35) [18] were separated into groups with mild (GS 0.35–1.49) and severe (GS 1.5–2.0) oesophagitis by using the median GS score value of 1.5. We excluded cases with lymphocytic [20], infectious or eosinophilic [21] oesophagitis in re-evaluation (TJK).

### Immunohistochemistry

For immunohistochemistry, formalin-fixed paraffin-embedded (FFPE) tissue specimens were subjected to high-temperature antigen retrieval in Tris-EDTA buffer for 15 min. Sections were immunostained with a Dako Autostainer (Dako, Copenhagen, Denmark). Primary antibodies were monoclonal mouse IgG1 anti-TLR2 (MAB0066, diluted 1:75; Abnova, Taipei City, Taiwan), mouse IgG2a kappa anti-TLR4 (H00007099-M02, diluted 1:1000; Imgenex, San Diego, CA) and monoclonal mouse IgG2A anti-FXR (Clone #A9033A, PP-A9033A-00, diluted 1:300; R&D Systems, Minneapolis, MN). Antibody detection was performed with the Dako Envision kit (Dako, Copenhagen, Denmark), with diaminobenzidine as the chromogen. Rabbit serum was used as a negative control.

TLR and FXR immunoreactivities were assessed by three independent researchers (TJK, MN, NV) blinded to the clinical data. In each biopsy, the squamous epithelium region that showed the most severe histopathological inflammatory change was selected for evaluation. For TLR2 and TLR4 assessments, the squamous epithelium was divided into basal and superficial regions, which were evaluated separately for staining intensity (range: 0–3) and the percentage of stained cells (0–100%) [11]. Nuclear and cytoplasmic staining were assessed separately for TLRs. Nuclear FXR was stained homogeneously throughout the epithelium; thus, we only assessed nuclear intensity (range: 0–3) and the percentage of stained nuclei in the field of view (0–100%). Individual estimates that differed by >1, for the intensity score, or >30%, for the percentage, were reconciled in a separate consensus meeting. For statistical analyses, we calculated a histoscore (0–300) for each receptor by multiplying the mean intensity (0–3) by the mean percentage (0–100%).

### In situ hybridization

For ISH, specimens from six (6) patients were included for TLR2 and four (4) patients for TLR4 analysis, most of these specimens represented both normal and inflamed oesophageal mucosa. Sections from formalin-fixed specimens were studied with the RNAscope 2.5 HD Reagent Kit (Red) for FFPE tissue (cat. no. 322360) and probes for human TLR2 and TLR4 (RNAscope® Hs-TLR2, cat. no 403111; RNAscope® Probe- Hs-TLR4, cat. no. 311281; Advanced Cell Diagnostics, Newark, CA, USA) according to the manufacturer’s instructions. For positive and negative controls, we used human UBC (cat. no: 310041) and bacterial DapB (cat. no: 310043) probes.

For quantification of the hybridization signals of TLR2 and TLR4, images representing normal squamous epithelium, squamous epithelium in mild and severe esophagitis, were taken. Numbers of dots/squamous epithelial cells were determined using Qupath (v0.2.3; https://qupath.github.io/), an open source image analysis platform with trainable image analysis algorithms [22] as detailed in Online Resource 1. In each specimen, dots/cell determinations were performed in all lesion types present, including normal squamous epithelium and oesophagitis. In addition, for comparison, the dot density was determined in the dense inflammatory cell infiltrate of lamina propria, if present.

### Statistical analysis

Statistical analyses were performed with SPSS Statistics 24.0 (IBM Corp., Armonk, NY). TLR2, TLR4 and FXR immunohistochemical expression levels were dichotomized into two equally sized groups of low and high expression, based on the median value. Due to the skewed histoscore distribution, we used Kruskal-Wallis test with Bonferroni correction to compare expression levels between groups of histological oesophagitis. We applied two-tailed Spearman’s rank correlations to evaluate correlations between immunostaining intensities in basal and superficial oesophageal epithelium and the degree of histological and endoscopic oesophagitis.

## Results

Patient demographic data are summarized in Table 1. The 84 included patients had a median age of 58 (range 20–94) years and 52% were women (*n*=44). Women had more often mild oesophagitis (61%) but men severe (63%). The re-evaluated cohort included 26 cases with histologically normal oesophagus, and 58 cases had reflux oesophagitis, according to the GS score (28 mild, 30 severe). Two patients were diagnosed with lymphocytic oesophagitis and were excluded from further analyses. There were no cases with eosinophilic esophagitis. Histological and endoscopic oesophagitis were intercorrelated (*p*< 0.01) (Table 3).

**Table 1:** Patient demographics

	Histological diagnosis			Endoscopic diagnosis		
Characteristic	Normal epithelium	Mild oesophagitis	Severe oesophagitis	Normal endoscopy	Mild oesophagitis	Severe oesophagitis
Age						
<30	13/26	3/28	3/30	6/38	2/16	2/30
30–60	13/26	14/28	13/30	19/38	5/16	14/30
>60	10/26	11/28	14/30	13/38	9/16	14/30
Sex						
Female	16/26	17/28	11/30	27/38	6/16	10/30
Male	10/26	11/28	19/30	11/38	10/16	20/30
LA class						
Normal	13/26	0/28	0/30	12/38	1/16	0/30
LA A-B	4/26	9/28	5/30	1/38	12/16	5/30
LA C-D	0/26	0/28	21/30	0/38	1/16	20/30
N/A	9/26	19/28	4/30	25/38	2/16	5/30

### TLR2, TLR4 and FXR expression in normal and inflamed oesophageal squamous epithelium

#### Immunohistochemistry

**Fig. 1:**
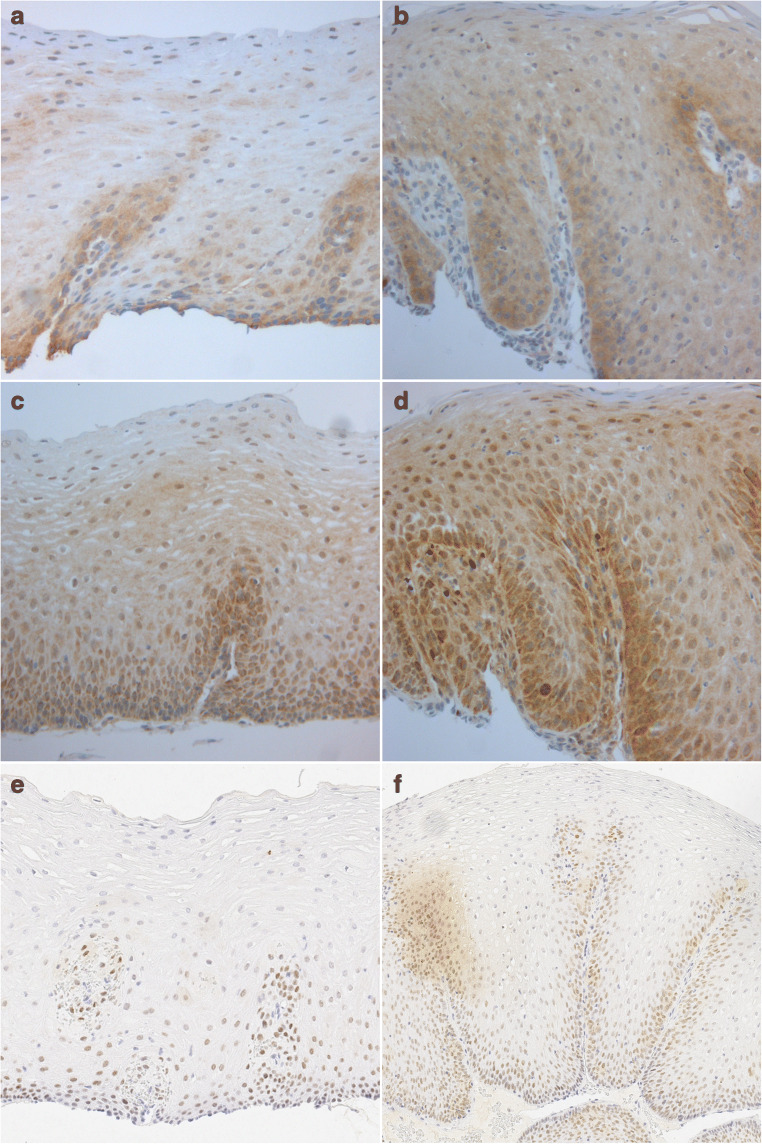
Receptor expression in oesophagitis. Immunohistochemical staining shows TLR2 (**a**, **b**), TLR4 (**c**, **d**) and FXR expression (**e**, **f**) in normal oesophageal squamous epithelium (**a**, **c**, **e**) and in oesophagitis (**b**, **d**, **f**). Sections are oriented as follows: top: superficial layer; bottom: basal layer

**Fig. 2:**
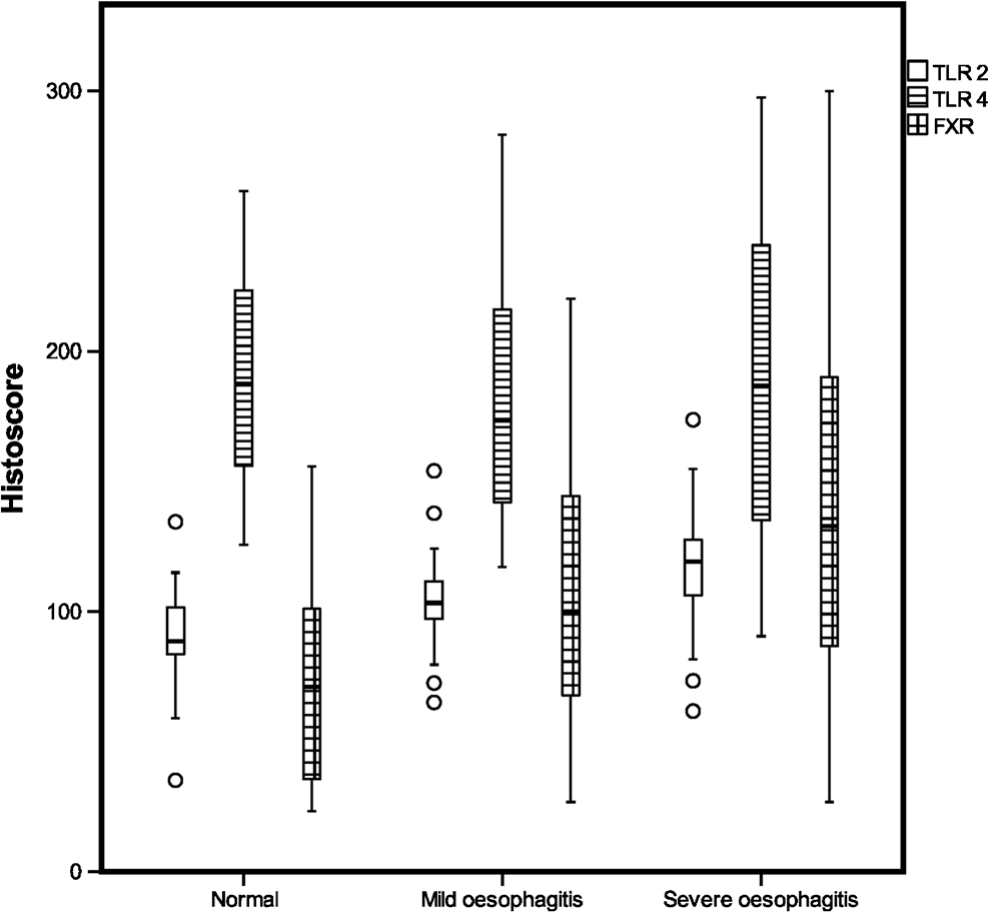
Histoscores show TLR2, TLR4 and FXR expression levels in different degrees of oesophagitis

TLR2, TLR4 and FXR were expressed throughout normal squamous epithelium and in oesophagitis (Table 2). In normal oesophageal epithelium, TLR2 was prominently expressed in the basal epithelium, with strong cytoplasmic staining and moderate nuclear staining. The stain gradually diminished towards the superficial layers where weak expression was observed in the nuclei and cytoplasm (Fig. 1). In oesophagitis, the intensity and histoscore of cytoplasmic TLR2 staining increased significantly in superficial layers, but the gradient of strong staining in the basal layer to weaker staining at the surface persisted. Similarly, in oesophagitis, nuclear TLR2 staining was significantly stronger in the superficial layer but similar in the basal layer, compared to non-inflamed epithelium (Figs. 1a–b, 2, Table 2).

**Table 2:** Expression of TLR2, TLR4 and FXR in normal oesophageal squamous epithelium and in mild and severe oesophagitis

Protein/location	Normal epithelium (histoscore)	Mild Esophagitis (histoscore)		Severe Esophagitis (histoscore)	
	Median (IQR)	Median (IQR)	*p*	Median (IQR)	*p*
TLR2					
Basal cytoplasm	133 (116–160)	187 (167-200)	**<0.05**	200 (187–241)	**<***0.01*
Basal nuclei	80 (67–90)	77 (60–93)		87 (70–97)	0.36
Superficial cytoplasm	73 (63–83)	80 (73–90)	0.29	90 (77–111)	**<***0.01*
Superficial nuclei	67 (60–67)	80 (63–87)		83 (62–88)	0.17
TLR4					
Basal cytoplasm	193 (150–193)	233 (200–267)		231 (137–278)	0.22
Basal nuclei	233 (210–233)	167 (120–260)		233 (122–285)	0.22
Superficial cytoplasm	139 (90–139)	93 (87–167)		120 (85–177)	0.90
Superficial nuclei	194 (180–194)	180 (160–210)		210 (170–255)	0.34
FXR	62 (36–62)	100 (47-147)	0.20	133 (92–195)	**<***0.01*

*p*-values represent the comparison with normal squamous epithelium. There were no significant differences between mild and severe esophagitis

*TLR* Toll-like receptor, *FXR* Farnesoid X receptor, *ns* not significant

TLR4 staining was more intense than TLR2 staining, in both normal and inflamed oesophageal epithelium. In many cases, the most basal layer was not stained, but suprabasal cells in the basal layer and the papillae showed strong nuclear and cytoplasmic TLR4 staining. Cytoplasmic TLR4 staining was less intense in the superficial layer than in the basal layer of the epithelium. In contrast, nuclear TLR4 staining was strong in both layers (Figs. 1c–d, 2, Table 2). In oesophagitis, both nuclear and cytoplasmic TLR4 staining intensities were similar to those observed in normal epithelium, in both the basal and superficial layers (Table 2).

Nuclear FXR was expressed homogeneously across the full thickness of normal squamous epithelium, with mild or moderate staining intensity. FXR staining intensity was significantly stronger in oesophagitis than in non-inflamed squamous epithelium (Figs. 1e–f, 2, Table 2).

#### In situ hybridization for TLR2 and TLR4

ISH signals for TLR2 and TLR4 were present in low numbers (TLR2: range 0–0.9 dots/cell; mean 0.11; median 0.06; TLR4: range 0–0.4 dots/cell; mean 0.05, median 0.03) in oesophageal squamous epithelium, however showing a clear difference in comparison with negative controls where the dots were completely absent (Online Resource 1; Figs. 1, 2, 3, 4). In comparison with lamina propria inflammatory cell infiltrates, composed mainly of mononuclear inflammatory cells (Online Resource 1; Fig. 3), the numbers of dots/cell in squamous epithelium were approximately 78% (TLR2) and 72 % (TLR4) lower.

For TLR2, the number of dots/cell in normal squamous epithelium tended to be higher in the lower half as compared with the upper half (Online Resource 1; Figs. 1, 2 and 5). In reflux oesophagitis, there was an increase in the number of dots/cell, which in the superficial half of the epithelium was significant (Online Resource 1; Figs. 2 and 5). For TLR4, no differences between upper and lower half or between normal squamous epithelium and oesophagitis appeared (Online Resource 1; Figs. 2 and 6).

### Relationships between receptor expression levels and features of oesophageal inflammation

The correlations between TLR2, TLR4 and FXR expression and the severities of histological and endoscopic oesophagitis are summarized in Table 3. We found that TLR2 expression, in both basal and superficial layers, was significantly correlated with both endoscopic and histological features of oesophagitis. TLR4 expression was not associated with endoscopic or histological oesophagitis features. FXR was positively correlated with histological oesophagitis features. TLR2 and TLR4 expression levels were not interdependent. However, FXR expression correlated significantly with TLR2 basal histoscore (*p* < 0.05).

**Table 3:** Correlations between TLR2, TLR4 and FXR expression and oesophagitis

	Basal TLR2	Superficial TLR2	Total TLR2	Basal TLR4	Superficial TLR4	Total TLR4	FXR	Macroscopic oesophagitis
Histological oesophagitis	0.45 (**<***0.01*)	0.34 (**<***0.01*)	0.48 (**<***0.01*)	−0.04 (0.76)	−0.11 (0.38)	−0.04 (0.76)	0.30 (**<***0.05*)	0.60 (**<***0.01*)
Basal TLR2		0.50 (**<***0.01*)	0.83 (**<***0.01*)	0.05 (0.71)	−0.07 (0.58)	0.05 (0.71)	0.29 (**<***0.05*)	0.33 (**<***0.01*)
Superficial TLR2			0.61 (**<***0.01*)	−0.07 (0.56)	−0.07 (0.56)	−0.01 (0.91)	0.19 (0.13)	0.25 (**<***0.05*)
Total TLR2				0.04 (0.72)	−0.13 (0.29)	−0.01 (0.91)	0.19 (0.13)	0.34 (**<***0.01*)
Basal TLR4					0.61 (**<***0.01*)	0.89 (**<***0.01*)	0.13 (0.32)	0.07 (0.58)
Superficial TLR4						0.72 (**<***0.01*)	0.23 (0.08)	−0.06 (0.62)
Total TLR4							0.23 (0.08)	0.07 (0.58)
FXR								0.22 (0.08)

Both histological and macroscopic (endoscopic) oesophagitis were divided into absent, mild and severe. Data are the correlation coefficient (*p*-value), calculated with 2-tailed Spearman correlation

*TLR* Toll-like receptor, *FXR* Farnesoid X receptor

TLR2 expression showed a trend for correlation (rho 0.22, *p*=0.059), and FXR expression showed a significant correlation (rho=0.27, *p*=0.027) with intraepithelial neutrophil scores. Our cohort included five patients with *H. pylori* infection based on histology: four in the oesophagitis group and one in the control group. Presence of *H. pylori* infection did not significantly associate with the expression of the studied markers

## Discussion

This study was the first to describe the expression of innate immunity receptors, TLR2 and TLR4, in the oesophageal squamous epithelium in a representative series of patients with and without reflux oesophagitis. By using immunohistochemistry and in situ hybridization, we showed that both TLR2 and TLR4 were expressed in normal oesophageal squamous epithelium, which indicated that both receptors were involved in normal oesophageal squamous epithelium physiology. In reflux oesophagitis, TLR2 expression significantly increased in superficial cells, but TLR4 expression did not change. The bile acid receptor, FXR, was weakly expressed throughout the epithelium in normal mucosa, and expression increased in oesophagitis. FXR expression was correlated with the basal TLR2 expression. These findings suggest that GERD develops as a multifactorial inflammatory condition, which involves innate immunity activation.

Our study showed predominantly superficial increase in TLR2 expression in reflux oesophagitis. The increase in TLR2 expression was consistent with the general pattern reported for TLR2 in inflammatory conditions [23, 24] and also with a study from Verbeek et al. [25], who showed that TLR2 mRNA expression increased non-significantly in reflux oesophagitis. The shift to more superficial distribution of TLR2 suggests that luminal TLR2 ligands might play a role in GERD pathogenesis. In contrast for TLR4, we observed both nuclear and cytoplasmic expression in all layers of normal squamous epithelium, but expression levels did not change in GERD. Only one previous study on oesophageal TLR4 expression by Verbeek et al. [26] has reported cytoplasmic TLR4 expression in basal cells of normal oesophageal squamous epithelium. Those authors found that in oesophagitis, TLR4 expression extended towards the epithelial surface, and that TLR4 mRNA levels increased in oesophagitis, but they provided no statistical data [26]. The discrepancy in these findings might be related to methodological issues, such as antibodies used or the sensitivity of immunostaining. In addition, the RT-PCR-based expression analyses of Verbeek et al. [26] did not differentiate between the cellular origins of the receptor; subepithelial inflammatory cells might have contributed to the observed increase in TLR4 mRNA expression in oesophagitis. A recent in vitro study found that both bile and acid exposure induced TLR4 mRNA expression in an oesophageal squamous cell line [27]. Since the cell line in question was transfected with SV40 virus and had several missing chromosomes, the line did not functionally represent normal oesophageal squamous cells. Altogether, these findings require further confirmation. Increase of both TLR2 and TLR4 has been reported in eosinophilic oesophagitis, however without clear cellular localization described [28]. Whether alterations in TLR2 and TLR4 expression levels have any specificity for the type of oesophagitis warrants additional studies.

Could different TLR2 and TLR4 expression levels in oesophagitis be associated with disease-related changes in the oesophageal microbiome? In healthy conditions, the oesophageal microbiome is similar to that of the oropharynx [29]. Normal microbiome is dominated by gram-positive bacteria [30, 31], and the major TLR2 ligands are expressed in the outer membrane of gram-positive bacteria [32]. During GERD, the microbiome shifts to harbouring more gram-negative and anaerobic species [30, 31]. TLR4 recognizes mostly gram-negative species [33]; however, TLR2 does also play a role in gram-negative infections, which probably in part explains the observed increase in TLR2 expression in oesophagitis [32]. The factors explaining the unchanged TLR4 expression in oesophagitis are less obvious. A potential rationale could be endotoxin tolerance, which might require downregulation of the TLR4-mediated inflammatory response to lipopolysaccharides (LPS), following short or sustained LPS exposure [34]. Alternatively, the inert TLR4 expression might be due to bacterial species-related differences in LPS structure and/or effects, since some LPS-related molecules can inhibit TLR4 response [35].

The downstream effects of TLR2 activation in oesophagitis remain speculative. In addition to downstream proinflammatory effects, TLR2 stimulation was shown to improve mucosal integrity and epithelial barrier preservation in oesophageal squamous epithelium [24], in ex vivo and in vitro model intestinal epithelial cell lines [36] and in Barrett’s oesophagus [25]. TLR2 activation has also induced epithelial proliferation [37] in the mucosa of small intestine. Thus, squamous epithelial hyperplasia, characteristic of reflux oesophagitis, could be related to TLR2 activation in prevention of mucosal injury.

We previously showed that TLR4 expression increased in the Barrett metaplasia-dysplasia-carcinoma sequence [11]. Here, we did not observe increased TLR4 expression in oesophagitis; however, we could not exclude TLR4 activation, since upregulation is not necessary to activate signalling [27]. Accordingly, the role of TLR4 requires further study.

We observed nuclear FXR expression throughout normal squamous epithelium. During oesophagitis, FXR expression was significantly upregulated with a homogeneous distribution. This finding was consistent with previous findings [15], which were based on only 6 patients/group. Our novel finding was the correlation between FXR expression and the basal TLR2 expression. FXR expression and superficial TLR4 (*p* = 0.08) and total TLR4 (*p* = 0.08) histoscores showed near significant trends for correlation. Therefore, our results confirmed the role of FXR in oesophagitis and support a link between FXR and TLR2 and possibly TLR4 responses. Previously, based on mouse studies [38, 39], it was proposed that FXR activation inhibited TLR4 signalling. Moreover, it has been suggested that bile acid activation of FXR and G-protein bile acid receptor-1 could reverse the pro-inflammatory cascade activated by TLR4. Among several potentially inhibitory pathways, FXR seems to repress primarily NF-kB-dependent gene expression. Lian et al. [16] showed that FXR knockout mice were highly susceptible to gastric ulcers, due to a lack of TNF-α suppression. Our observations suggested that, during GERD, protective FXR and TLR2 receptors were robustly upregulated, potentially via common regulation. The proinflammatory TLR4 receptors remained inert, possibly due to FXR activation, but our results could not confirm the correlation between TLR4 and FXR.

This study had some limitations. First, the sample size was fairly small. However, many studies in this field had smaller series, due to their pilot nature. Second, we collected data without considering gender or age. Therefore, we could not assess the influence of these factors. In the future, it would be interesting to evaluate downstream TLR and FXR signalling pathways and the potential role of FXR in TLR4 downregulation. Future studies might also address mechanisms of gender-related differences and the potential roles of menopausal status and oestrogen derivatives in GERD development.

## Conclusions

We showed that TLR2 and FXR were strongly upregulated, but TLR4 was unchanged during reflux oesophagitis. Our finding that TLR2 and FXR were correlated supports the notion that FXR could modulate innate immunity responses. Clearly, in reflux esophagitis, the factors that contribute to acid- and bile-mediated tissue damage and mucosal injury are complex and require further investigation.

## Supplementary information

Online Resource 1. Description of the in situ hybridization protocol for TLR2 and TLR4 in oesophageal specimens and the representation of TLR2 and TLR4 mRNA expression in normal oesophagus and in reflux oesophagitis. (DOCX 6953 kb)

## Data Availability

The datasets generated during and/or analysed during the current study are available from the corresponding author on reasonable request.
