# Supplementary material for: Pathophysiology of reflux oesophagitis: role of Toll-like receptors 2 and 4 and Farnesoid X receptor
Source: Virchows Arch. 2021 Mar 8;479(2):285–93. doi: 10.1007/s00428-021-03066-w (PMC8364528; doi:10.1007/s00428-021-03066-w)
Supplement: Supplementary file 1 — Description of the in situ hybridization protocol for TLR2 and TLR4 in oesophageal specimens and the representation of TLR2 and TLR4 mRNA expression in normal oesophagus and in reflux oesophagitis. (DOCX 6953 kb) [file 428_2021_3066_MOESM1_ESM.docx]

**Supplementary file**

**Pathophysiology of reflux oesophagitis: role of Toll-like receptors 2, 4, and Farnesoid X–receptor**

Minna Nortunen, Nina Väkiparta, Juha Saarnio, Tuomo J. Karttunen, Heikki Huhta

1. Determination of dots/cell counts in situ hybridization (ISH) for TLR2 and TLR4 in oesophageal specimens

As presented in Materials and methods -section, we used RNAscope 2.5 HD Reagent Kit (Red) for FFPE tissue (cat. no. 322360) and probes for human TLR2 and TLR4 (RNAscope® Hs-TLR2, cat. no 403111; RNAscope® Probe- Hs-TLR4, cat. no. 311281; Advanced Cell Diagnostics, Newark, CA, USA), with fast red as chromogen and haematoxylin for background staining. For determination of number of mRNA dots in oesophageal tissues we used Qupath (v0.2.3; https://qupath.github.io/), an open source image analysis platform [22]. Numbers of dots were separately quantitated in upper and lower half of squamous epithelial cells, and for comparison, also in the inflammatory cell infiltrates.

In the determination of dot counts we first tested subcellular detection option in Qupath (Analyze 🡪 Cell detection 🡪 Subcellular detection). Before subcellular detection, stain channel for ISH label (fast red) was determined by annotating a representative dot, followed by detection of stain vector by Qupath. Although positive dots were successfully detected by the subcellular detection algorithm, also foci, which by visual assessment were negative were detected. We were not able to adjust settings not to include these clearly invalid foci, such as strongly haematoxylin stained cell membranes occasionally present at the edges of specimens. Therefore we tested pixel classifier algorithm, which, after training with relevant annotations, appeared to reliably detect the spots. Performance of the detection system was assessed for all analysed images by visually confirming that only relevant dots were detected.

Our final workflow using the pixel classifier had three steps: A) pre-processing; B) annotation of representative areas of upper and lower half of oesophageal squamous epithelium for measurement of dot and cell counts; C) determination of dot counts with pixel classifier; D) determination of cell counts with cell detection algorithm of Qupath.

1. In the preprocessing step, type of image was determined as Brightfield other, and stain vectors were adjusted (Analyze 🡪 Preprocessing 🡪 Estimate stain vectors 🡪 Auto detect).
2. Representative areas of lower and upper half of squamous epithelium were annotated using rectangle tool or brush tool or their combination. To get comparative expression, data regions composed of heavy lamina propria inflammatory cell infiltrate, were additionally annotated.
3. In quantification of dots we used pixel classification option. For training of pixel classifier, several positive dots in several images were annotated, and similarly, areas in the epithelium without any positive dots were annotated. To optimize classifier, options available in the setup of classifier were tested by comparing performance of the classifier with visual detection of the dots (Figure 1). Along with such testing of the performance of the classifier, following options were finally used: Classifier: Random trees; Resolution: high downsample; Default multiscale features: Channels: red, green, blue, haematoxylin, residual; Scales 1.0; Features: Gaussian, Structure tensor min eigenvalue; Local normalization: Local mean & variance. After the detection step, Create objects option in the Pixel classifier was used to get total number of dots detected.
4. For counting of squamous epithelial cells, cell detection option of Qupath was used (Figure 1), after parameters of this algorithm were optimized to include most of the cells within the annotations.

Finally, dots/cell and dots/areal unit for upper and lower half of squamous epithelium, inflammatory cell infiltrate and smooth muscle cells were counted.

1. TLR2 and TLR4 mRNA expression in normal oesophagus and in reflux oesophagitis

ISH preparations with specific probes for TLR2 and TLR4 mRNA showed dots in squamous epithelial cells and in inflammatory cells forming collections in lamina propria (Figures 2-3). In contrast, ISH with negative control probe showed no dots (Figure 4). By using pixel classification algorithm built in the image analysis program Qupath it was possible to reliably and objectively quantitate dots (Figure 1). The cell detection algorithm in Qupath provided the number of cells (Figure 1) for the calculation of the number of dots/cell.

For TLR2, the number of dots/cell in squamous epithelium varied from 0 to 0.9 (mean 0.11; median 0,06; Figure 5) and for TLR4 from 0 to 0.4 (mean 0.05, median 0.03; Figure 6). Providing an internal positive control, dots were clearly more abundant in inflammatory cell infiltrate, where numbers of dots/cell for TLR2 varied from 0.25 to 0.95/cell (mean 0.52, median 0.45), and those for TLR4, from 0.06 to 0.42 (mean 0.18; median 0.12).

In normal squamous epithelium, the number of dots for TLR2 tended to be more abundant in the basal half of epithelium as compared with the upper half (Figure 5). In oesophagitis, number of the dots tended to increase in both upper and lower half of the epithelium (Figures 2 and 5), increase in the upper half showing evidence for statistical significance in comparison of both mild and severe oesophagitis to normal epithelium (p<0.033, Kruskal-Wallis test).

Numbers of dots for TLR4 were low both in upper and lower half of normal squamous epithelium (Figure 6), and no apparent increase was observed in oesophagitis (Figure 6).

**References**

22. Bankhead, P., Loughrey, M. B., Fernández, J. A., Dombrowski, Y., McArt, D. G., Dunne, P. D., et al. (2017). QuPath: Open source software for digital pathology image analysis. *Scientific Reports, 7*(1), 16878-017.


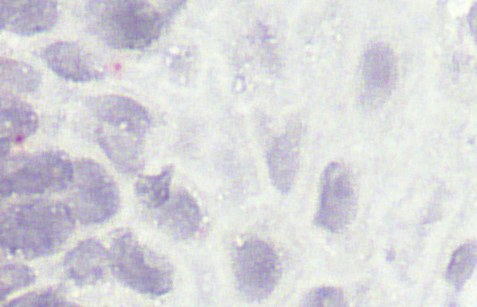

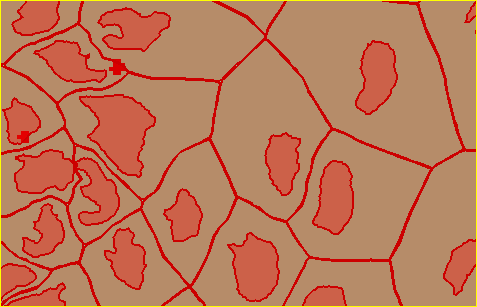


A B

**Figure 1**. Determination of dots/cell in ISH for TLR2. A. Image showing a field of the basal part of esophageal squamous epithelium with a few positive dots (arrows). B. Same field after detection of the dots and cells with Qupath pixel classification and cell detection algorithms, respectively. ISH for TLR2, fast red chromogen; hematoxylin counterstaining.


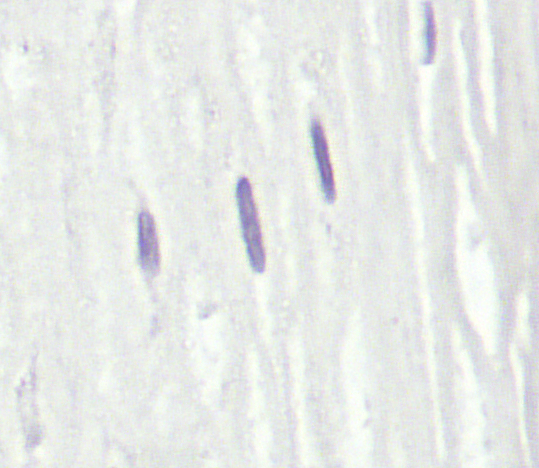

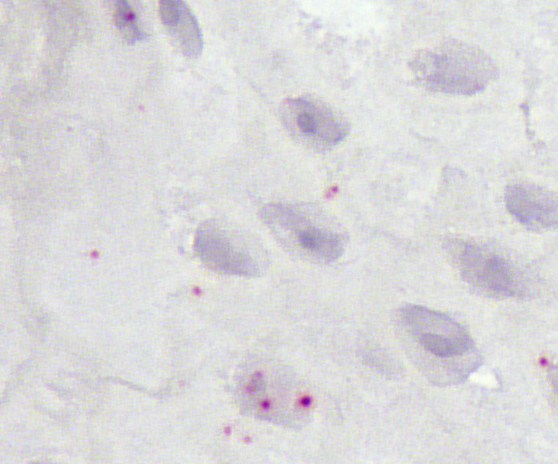


A B


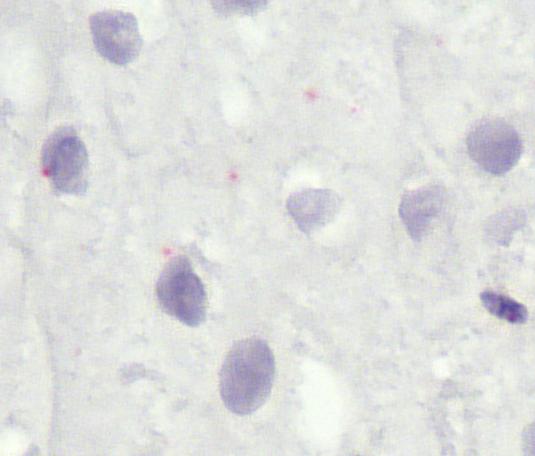

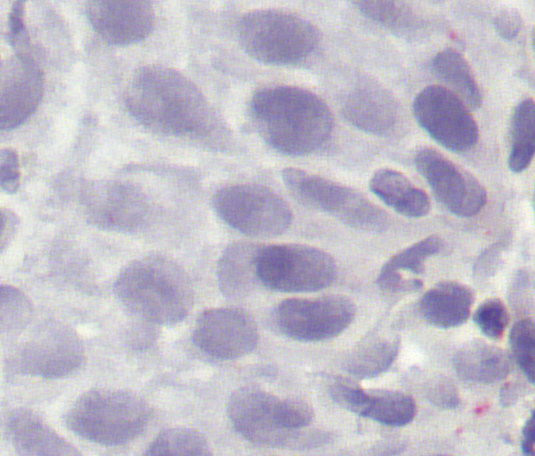


C D

**Figure 2**. Examples of ISH for TLR2 (A; B) and for TLR4 (B, C) in normal and inflamed esophagus. TLR2. Upper half of oesophageal squamous epithelium shows no dots in normal esophagus (A), while red dots indicating presence of mRNA (arrows) are present in the cells of upper half of squamous epithelium in reflux oesophagitis (B). TLR4. Few red dots are seen in oesophagitis in both upper (C) and lower (D) part of the oesophagus (arrows).


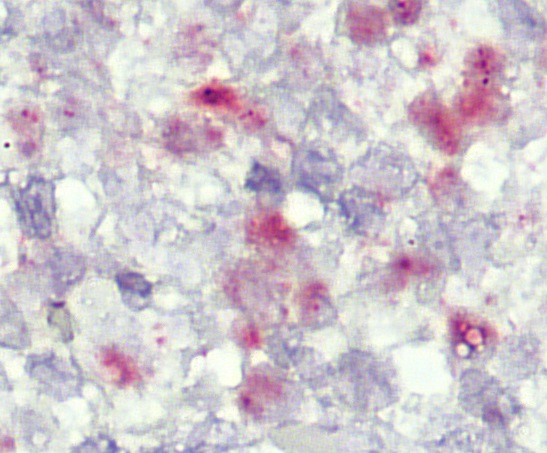

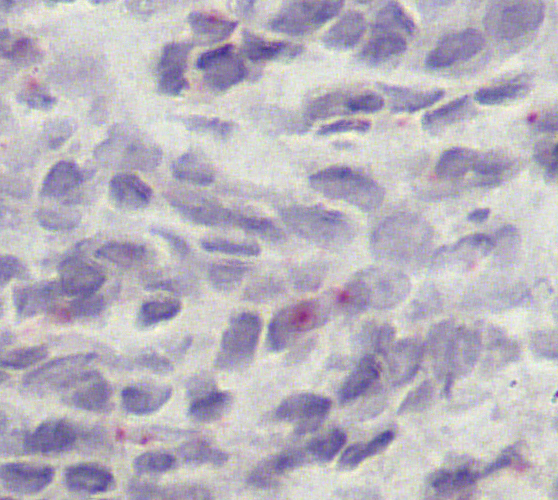


A B

**Figure 3**. Examples of ISH for TLR2 and TLR4 in heavy inflammatory cell infiltrate in oesophagitis. Abundant red dots for TLR2 (A) and a moderate abundance of red dots for TLR4 are seen.


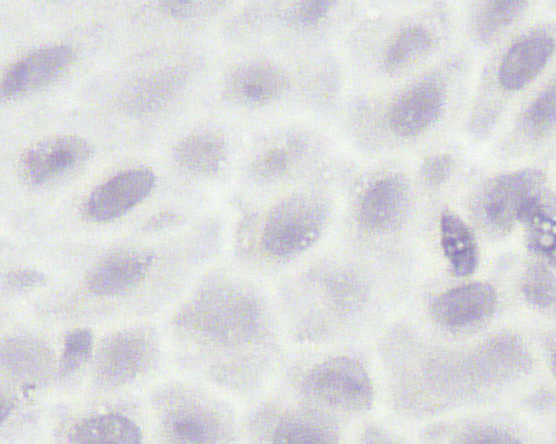

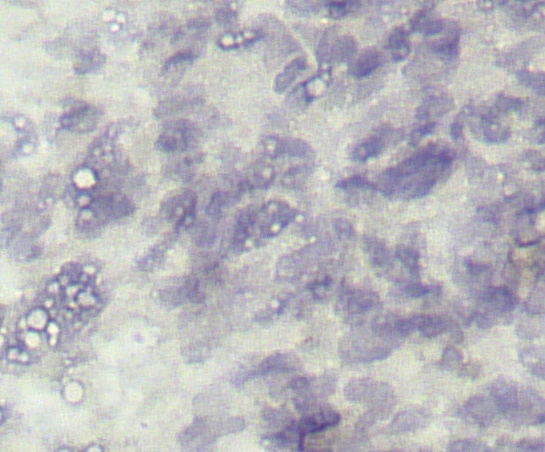


A B

**Figure 4**. Negative control ISHs with an irrelevant probe show no labelling in oesophageal squamous epithelium (A) or in heavy inflammatory cell infiltrate in reflux oesophagitis (B).


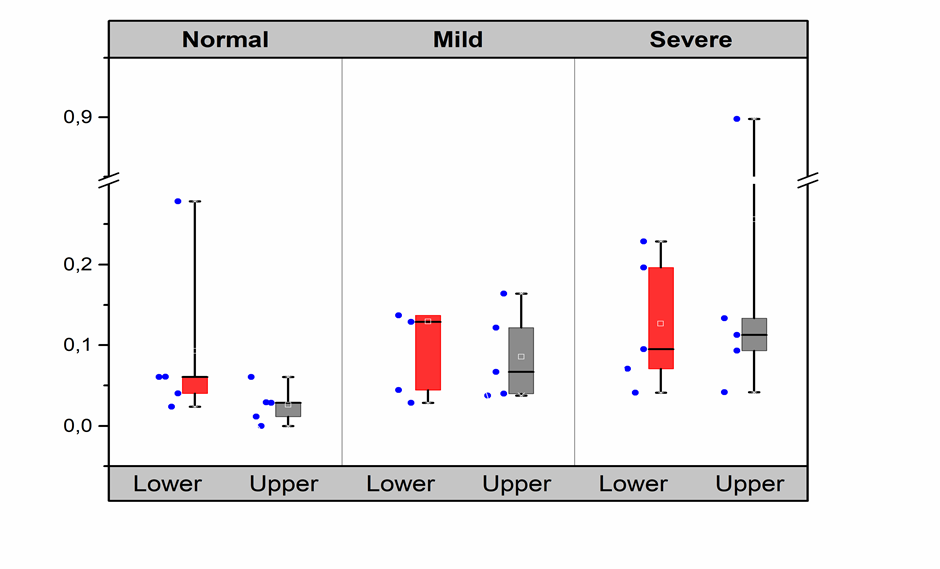
**Figure 5.** Scatter and box plot showing distribution of TLR2 dot counts/cell in upper and lower half of normal oesophageal squamous epithelium and in mild and severe reflux oesophagitis. Box indicates 25-75 percentile, whiskers show range, horizontal line indicates median, and white box mean.



**Figure 6.** Scatter and box plot showing distribution of TLR4 dot counts/cell in upper and lower half of normal oesophageal squamous epithelium and in mild and severe reflux oesophagitis. Box indicates 25-75 percentile, whiskers show range, horizontal line indicates median, and white box mean.
